# PERI_DEP: A dataset of mother's mental health in Pakistan

**DOI:** 10.1016/j.dib.2025.111621

**Published:** 2025-05-07

**Authors:** Amna Zafar, Beenish Ayesha Akram, Muhammad Wasim, Maham, Ivan Miguel Pires, Paulo Jorge Coelho

**Affiliations:** a Department of Computer Science, University of Engineering and Technology, Lahore, Pakistan; b Department of Computer Engineering, University of Engineering and Technology, Lahore, Pakistan; c Department of Computer Science, University of Management and Technology, Lahore (Sialkot Campus), Pakistan; d Instituto de Telecomunicações, Escola Superior de Tecnologia e Gestão de Águeda, Universidade de Aveiro, Águeda, Portugal; e School of Technology and Management, Polytechnic University of Leiria, Leiria, Portugal; f Institute for Systems Engineering and Computers at Coimbra, University of Coimbra, Coimbra, Portugal

**Keywords:** Perinatal, Prenatal, Postnatal, Depression, Mental health

## Abstract

Perinatal depression (PND) represents a multifaceted mental health issue that impacts women throughout the perinatal period. Existing datasets have a class imbalance issue, resulting in biased outcomes. In Pakistan, we developed a novel dataset called PERI_DEP. This dataset leverages the Patient Health Questionnaire (PHQ-9), Edinburgh Postnatal Depression Scale (EPDS), and socio-demographic questionnaires to gather information about mental health state and socioeconomic details of women participants in urban and rural areas. Our novel PERI_DEP dataset contains 14,008 samples, and women from Lahore and Gujranwala participated. To tackle the issue of class imbalance, we employed Generative Adversarial Network (GAN) oversampling technique on our data. A key insight derived from this dataset is the comparative socio-demographic divide of women in rural and urban areas of Pakistan. PERI_DEP dataset enhances hospital capabilities by streamlining screening processes, customizing interventions, and enabling researchers to identify risk factors and develop new treatments accurately.

Specifications table

**Table:** 

Subject	Health and Medical sciences
Specific subject area	Analysis of Perinatal Depression in Urban and Rural Areas of Pakistan using PHQ-9, EPDS and Socio-demographic Data
Data format	Raw, Analysed, Filtered
Type of data	2 processed tables (CSV format) and 1 Jupyter notebook file
Data collection	The data were collected from June to November 2023. Participants were recruited through a convenience sampling approach at private obstetrics and gynaecology clinics near Gujranwala for rural data and at the outpatient department of obstetrics and gynaecology in Lahore. Standard mental health assessment tools including Patient Health Questionnaire (PHQ-9) and Edinburgh Postnatal Depression Scale (EPDS), along with custom socio-demographic questionnaire were used to gather data on mental health and socio-economic conditions of pregnant women and new mothers
Data source location	Country: Pakistan
Data accessibility	Repository Name: PERI_DEP Dataset DOI: 10.5281/zenodo.11094957 Direct URL to the data: https://zenodo.org/records/11403247

## 1 Value of the Data

• PERI_DEP dataset addresses class imbalance problem found in existing studies on perinatal depression using data augmentation techniques. For this purpose, we have applied GAN oversampling technique to create synthetic samples derived from the original dataset which ensures balanced and reliable outcomes. The original dataset contains 14008 records from pregnant women and new mothers during their perinatal period. The number of records labelled depressed were in higher number compared to non-depressed class. After applying GAN oversampling, the number of instances of non-depressed class increased, creating a balanced dataset.
• The PERI_DEP dataset contains mental health records of pregnant women and new mothers from rural and urban areas. An in-depth analysis of the dataset provides key insights about impact of rural vs. urban socio-economic divide on perinatal depression cases. The findings from the dataset reveals that a large proportion of Pakistani women included in the study sample are facing mental health problems. Several factors are found to be linked with mental stress of the women, including young maternal age, little or non-existent family support, lower financial state and increasing preference for male child especially in rural areas due to societal and family pressure, lack of education and cultural barriers.
• This study provides useful insight for public health policy makers and medical practitioners to develop intervention programs for improvement in mental health of pregnant women and new mothers. Screening programs for pregnant women should be implemented by maternity clinics for timely detection of those with symptoms of perinatal depression. This should be followed by the provision of appropriate counselling and treatment.
• The performance of the machine learning models are affected by imbalanced training data, producing biased results. This work solves the problem of class imbalance by applying GAN oversampling technique. Researchers can reuse the balanced PERI_DEP dataset to develop and test advanced machine learning models for accurate detection of perinatal depression. Furthermore, this dataset is useful for researchers in public health domain, policy makers and medical doctors in developing efficient intervention programs for early detection and diagnosis of perinatal depression among pregnant women and new mothers especially in a developing country like Pakistan.

## 2 Background

Mental health issues during the perinatal period can result in numerous health challenges for both the mother and her child. A comprehensive meta-analysis of 43 studies shows that combined depression rates during pregnancy and after delivery are 37% and 30%, respectively, in Pakistan [1]. In the research article [2], the authors conducted a study to develop a data set on postnatal depression. They applied machine learning methods to detect postnatal depression. However, due to class imbalance issue in the dataset, biased results were generated affecting the performance of the machine learning model used. In another study [3], the researchers developed a data set focusing on women aged 19 to 32 potentially, overlooking those at high risk of postnatal depression due to selection bias. Factors contributing to this bias are exclusion of illiterate women and preference for healthier women to participate. The authors in research article [4] developed a dataset by surveying 261 new mothers suffering from postnatal depression using EPDS questionnaire. However, as the size of the dataset is small, it is insufficient to train advanced machine learning models for automatic detection of perinatal depression.

In research work [5], the authors conducted an online survey of pregnant women to develop a dataset. However, they relied on participants to provide accurate data, which introduces risks of cognitive and recall biases. The research used anonymous surveys and previously validated tools to mitigate these biases. In another study conducted in Taiwan [6], surveys were conducted from 156 women during their pregnancy period. The authors employed Perceived Stress Scale, Centre for Epidemiologic Studies Depression scale, and State-Trait Anxiety Inventory to measure stress, symptoms of depression, and anxiety. However, limited sample size was used with a 46.8% attrition rate, along with its exclusive focus on metropolitan areas and reliance on self-reported data instead of diagnostic interviews, which may affect accuracy and scope of its findings.

A study conducted in Bangladesh was based on socio-demographic questionnaire along with updated questions from EPDS, PHQ-2 for screening postpartum depression [7]. However, the study is limited due to selection bias in the data set and an inadequate sample size. The authors in [8] conducted a study on postpartum depression using 28,755 records from Pregnancy Risk Assessment Monitoring System during 2012–2013. However, considerable amount of socioeconomic and lifestyle data is missing from 28,755 of 72,540 participants, creating doubt on whether the findings accurately represent those who did not respond.

In a study in Pakistan [9], the researchers collected data from 500 pregnant women and new mothers during their perinatal period. However, their analysis of risk factors was restricted to Lahore only, due to limited data access, thereby constraining the relevance of the model to a broader audience without further study. To overcome the issues discussed in existing datasets, this work aims to develop a well-balanced dataset that generates reliable and accurate results.

Here are the significant contributions of this work:

• We have developed a novel dataset named PERI_DEP using PHQ9, EPDS, and socio-demographic questionnaires. The surveys were carried out from pregnant women and new mothers residing in urban and rural areas of Pakistan. The original PERI_DEP dataset contains 14,008 records.
• To solve the class imbalance, we have applied data augmentation using Generative Adversarial Networks (GAN) oversampling technique on 80% of the data. PERI_DEP dataset contains 14,586 samples after applying data augmentation technique.

## 3 Data Description

All data records are available as CSV files on the Zenodo data repository @ https://zenodo.org/records/11403247. The "dataset.csv" file contains 14,008 original samples, and the "augmented_data.csv" file contains 14,586 samples with almost equal number of depressed and non-depresses samples. In the "augmented_data.csv" file, column "Sufficient Money for basic needs" is a target variable used for GAN oversampling. Therefore, datatype of this column is numeric, which differs from the corresponding column in the "dataset.csv" file. A scaling rate of 10 or higher is labeled as depressed.

### 3.1 Analysis of PERI_DEP Dataset

The analysis provides insights into the prevalence and characteristics of depression symptoms among the study population. These findings will be crucial for understanding scope of the problem and development of appropriate interventions and support mechanisms for perinatal depression (PND).

Table 1 shows data from participants reveal a demographic primarily comprised of young women in their perinatal period, with the largest age group being 25–30 years (48.90%). Education levels vary, with a significant number being illiterate (17.81%) and the majority having achieved education up to the enrollment (28.11%) and intermediate (22.30%) levels. A vast majority (74%) of these women are not working, indicating a high prevalence of homemakers. Financially, more participants report their status as bad or average (57.31%) than good or very good (42.69%). Family planning choices are diverse, with a considerable number of women having one or fewer children (35.89%) and a majority being multigravida (73.99%), suggesting previous multiple pregnancy experiences. Husband's education spans a wide range, from illiteracy (22.85%) to higher education (4.75%). The preference for a joint family system (66.78%) contrasts with a predominantly poor relationship with family (64.50%), indicating potential social support issues. Miscarriages were reported by 32.21%, highlighting perinatal care concerns. Acceptance of current appearance is almost evenly split, with a slight majority expressing dissatisfaction (51.37%), and a substantial preference for male offspring (75.72%) reflects deep-rooted societal gender biases.

**Table 1:** Summary of participants characteristics.

Variable	Value (%)
Age in Years	
18-19	3.90
20-24	22.56
25-30	48.90
31-34	13.06
35-40	10.09
41-44	1.3
45-49	0.18
**Education**	
Illiterate	17.81
Primary to middle	25.84
Matriculation	28.11
Intermediate	22.30
Graduation to masters	5.94
**Current Occupation**	
Working	26
Not working	74
**Financial Status**	
Bad or average	57.31
Good or very good	42.69
**Number of Children**	
≤ 1	35.89
Two children	23.71
Three children	22.12
≥ 4	18.28
**Gravida**	
Primigravida	26.00
Multigravida	74.00
**Husband’s education**	
Illiterate	22.85
Primary to Middle	28.06
Matriculation	26.09
Intermediate	18.25
Graduation to Masters	4.75
**Family system**	
Nuclear	33.22
Joint	66.78
**Relationship with in-laws**	
Good	14.14
Moderate	21.36
Poor	64.50
**Previous Miscarriages**	
Yes	32.21
No	67.79
**Acceptance of the current appearance**	
No Acceptance	61.37
Fully Acceptance	48.63
**Male Gender Preference**	
Yes	75.72
No	24.28

**Fig. 1:**
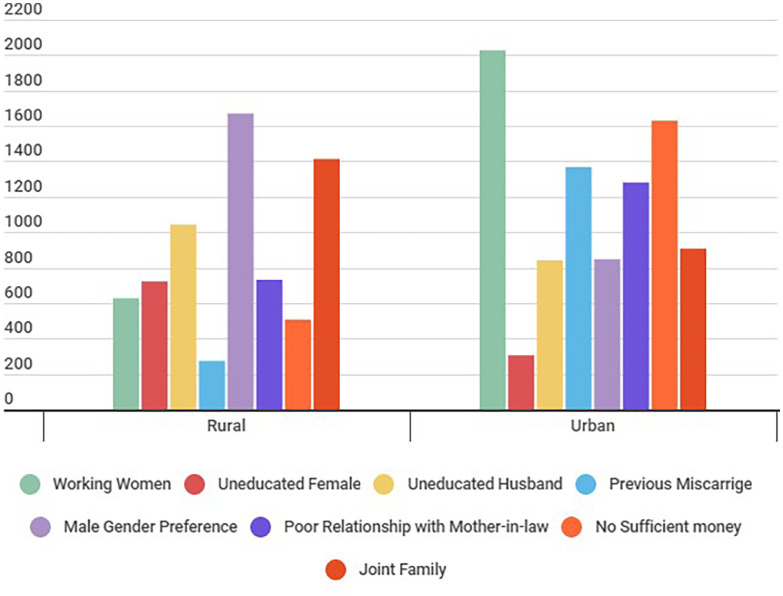
Socio-demographic comparison between urban and rural participants.

Fig. 1 compares urban and rural datasets, including working women's status, education of husband and female participants, miscarriage factors, child male gender preference, poor relationship with mother-in-law, joint family system, and financial status. The analysis shows that money is not enough to meet basic family needs. The working status of women is higher in urban areas than in rural areas. Child male gender preference is higher in rural areas as compared to urban areas. In rural areas, the relationship between participants and their mothers-in-law is weaker than in urban areas. Most families in both rural and urban areas are joint families. The miscarriage rate is high in urban areas. Women's literacy rate is higher than men in both areas.

## 4 Experimental Design, Materials and Methods

### 4.1 Dataset description

Our PERI_DEP dataset consists of records completed by pregnant women and new mothers in their perinatal period. Out of 14,008, 12,100 records are filled in urban areas and 1908 in rural areas. Utilizing GAN oversampling technique on 80% of the data, the dataset has expanded to 14,586 samples. This large and valuable dataset is used to study and find risk factors of PND among women.

### 4.2 Study design, and sampling

The primary method used for data collection in this study involved using standard mental health assessment tools i.e., EPDS and PHQ-9 tests along with socio-demographic survey. For the study, the population consists of pregnant women and new mothers living in rural and urban areas of Pakistan who met the inclusion criteria. The study recruited participants who expressed interest and availability for participation. For perinatal data collection in the rural areas, the study was conducted at the private obstetrics and gynecology clinics near Gujranwala from June to the end of July 2023. For data collection in urban areas, the study was conducted at the outpatient department of obstetrics and gynecology in Lahore from August to the end of November 2023. The institutional review board at the Services Institute of Medical Sciences (Pakistan) approved this study with the reference IRB/2023/1155/SIMS.

### 4.3 Ethical considerations

To carry out the study, we have recruited eligible participants. They were explained the study's purpose and assured that their confidentiality would be maintained. Written informed consent was obtained from the participants. Because participation was optional, it assumed that participants provided honest responses. The confidentiality of the participants' data must be protected. The data must be kept secure, and it should not be shared with anyone who is not involved in the study.

### 4.4 Survey instruments

We have used standard psychological assessment tools (PHQ-9, EPDS) and validated Likert scale to carry out the survey. To collect data about socio-economic conditions of the pregnant women and new mothers in urban and rural areas of a developing country like Pakistan, we have developed a new socio-demographic questionnaire. To the best of our knowledge, this is the first study on perinatal depression which combines standard psychological assessment tools (EPDS, PHQ-9) with a custom socio-demographic survey to capture the impact of socio-economic conditions on mental health of pregnant women and new mothers in a developing country like Pakistan.

**PHQ-9:** The interpretation of PHQ-9 scores provides a valuable guideline for assessing the severity of depression. The total score is calculated by summing the scaled responses on each question. This standardized assessment tool assigns different depression severity levels to specific score ranges. The PHQ-9 depression screening tool assesses nine symptoms of depression with 4 choices of response for each question. The responses are measured using Likert scale from 0-3. Here, 0 represents minimum impact and 3 represents high severity of the symptom. The total score is calculated by adding individual scores for all questions. The PHQ-9 cut-off score for depression is 10. A score of 10 or higher suggests that the respondent may be depressed [10].

**EPDS:** EPDS is a commonly utilized questionnaire to measure symptoms of depression in new mothers during postnatal period [11]. However, it has also been applied to evaluate presence and severity of depression in pregnant women during prenatal period. The EPDS consists of 10 short questions with four answer choices. For EPDS, based on the increased severity of depressive symptoms, the response categories are scored 0, 1, 2 and 3. The question number 3, 5-10 are scored in reverse order (i.e., 3, 2, 1, and 0). The total score is calculated by adding scores for each of the questions, ranging from 0 to 30, with higher scores indicating more severe depressive symptoms. Participants with a score of 13 or above are likely to be experiencing high-risk depression.

During our data collection phase, we used three questionnaires (EPDS, PHQ-9 and Sociodemographic). Most of the questions in both EPDS and PHQ-9 forms are similar. Therefore, in data compilation phase, we have mapped similar questions in both questionnaires into composite variables. We have used Likert scale to measure responses on both questionnaire. For each pair of mapped questions from both questionnaires, the responses given by the participants were averaged and rounded-up to generate a single value for every composite variable. For example, the first question on EPDS form is

EPDS1: I have been able to laugh and see the funny side of things:

■ As much as I always could
■ Not quite so much now
■ Definitely not so much now
■ Not at all

The above question is scored 0, 1, 2 or 3 with top box scored as 0 and the bottom box scored as 3. Here 0 represents positive behaviour with minimum impact of depressive symptom and 3 represents the maximum impact. This question is mapped on question number 1 on PHQ-9 form.

PHQ1: Little interest or pleasure in doing things

■ Not at all
■ Several days
■ More than half the days
■ Nearly Every Day

This question is also scored 0,1,2,3 with top-box scored 0 representing minimum impact and bottom box scored 3 which represents maximum severity of the depressive symptoms on the patient mental health. Therefore, if a participant has marked 3 on EPDS1 and also marked 3 on PHQ1. The average score of the participant will be 3 after mapping EPDS1 and PHQ1. Similarly, all EPDS questions have been mapped to similar questions in PHQ-9 form. Table 2 gives details of the mapping of 10 EPDS questions to similar questions in PHD-9 form.

**Table 2:** Mapping of EPDS and PHQ-9 Questions

EPDS	PHQ-9	Composite Variables
1. I have been able to laugh and see the funny side of things:	1. Little interest or pleasure in doing things	EPDS1-PHQ1
2. I have looked forward with enjoyment to things: As much as I ever did	4. Feeling tired or having little energy	EPDS2-PHQ4
3. I have blamed myself unnecessarily when things went wrong	6. Feeling bad about yourself — or that you are a failure or have let yourself or your family down	EPDS3-PHQ6
4. I have been anxious or worried for no good reason:	2. Feeling down, depressed, or hopeless	EPDS4-PHQ2
5. I have felt scared or panicky for no good reason:	8. Moving or speaking so slowly that other people could have noticed? Or the opposite — being so fidgety or restless that you have been moving around a lot more than usual	EPDS5-PHQ8
6. Things have been getting to me:	7. Trouble concentrating on things, such as reading the newspaper or watching television	EPDS6-PHQ7
7. I have been so unhappy that I have had difficulty sleeping	3. Trouble falling or staying asleep, or sleeping too much	EPDS7-PHQ3
8. I have felt sad or miserable:	2. Feeling down, depressed, or hopeless	EPDS8-PHQ2
9. I have been so unhappy that I have been crying.	2. Feeling down, depressed, or hopeless	EPDS9-PHQ2
10. The thought of harming myself has occurred to me.	9. Thoughts that you would be better off dead or of hurting yourself in some way.	EPDS10-PHQ9

**Socio-demographic Questionnaire**: For this study, we have developed a new socio-demographic questionnaire to record socio-economic status of pregnant women and new mothers. The socio-demographic questionnaire contained questions about age, education, working status, financial situation, and number of children, body shape change, husband education, and relationship with mother-in-law, family system, gestational weeks, gravida, and history of previous miscarriages. This information was calculated after taking informed consent. Questions like, Sufficient money for basic needs?; Acceptance of the current appearance?; Previous miscarriage?; were to be answered by choosing either "yes" or "no".

### 4.5 Data collection procedure

**Fig. 2:**
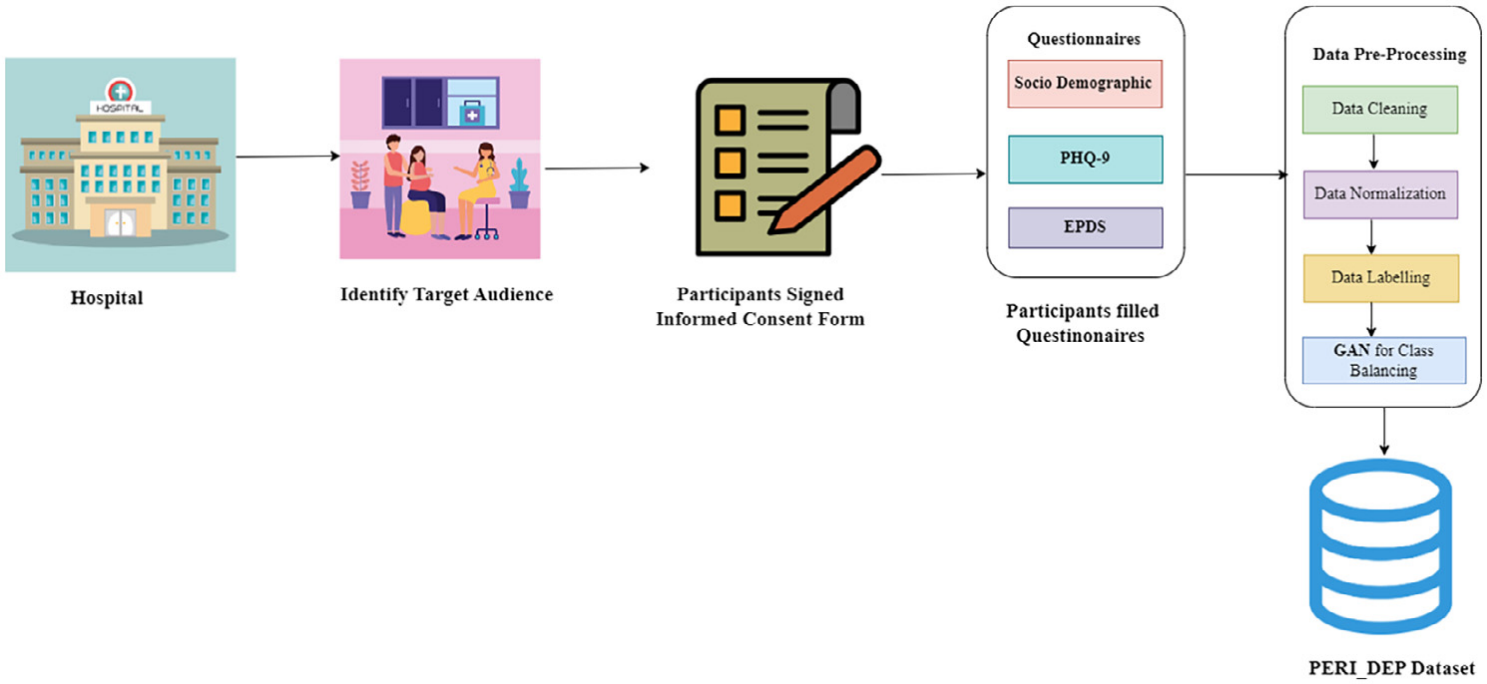
Complete data collection process.

The data was collected from the first week of June to the end of November 2023. Participants were recruited through a convenience sampling approach. The institutional review board at the Services Institute of Medical Sciences, Lahore (Pakistan), approved this study; the approval reference is IRB/2023/1155/SIMS. Participants signed an informed consent form. All participants received standardized training and instructions. Questionnaires were given to the participants. Completed questionnaires were reviewed for accuracy, and any inconsistent or unusual responses were identified, discussed, and corrected. All the information collected through questionnaires was entered into the Excel sheet daily. Fig. 2 shows the complete data collection process.

### 4.6 Inclusion criteria

Women included in the study are those who met the following criteria:

• Able to read or understand in English, Urdu, or Punjabi;
• Having Pakistani nationality;
• Willing to participate in the survey;
• Had no known mental health conditions;
• Participants are women aged 18 or above;
• Participant who are in their 2nd or 3rd trimester and have gestational age above 26
• Eligible participants include all primigravida and multigravida mothers.

### 4.7 Exclusion criteria

• For perinatal data collection, women under the age of 18 were excluded for ethical reasons;
• Women who do not speak English, Urdu, or Punjabi were excluded from the study;
• Women who were taking medications that could confound the study's results were excluded;
• Women who experience a high-risk pregnancy were also excluded from the study;
• Female participants with medical disorders, particularly those connected to trauma, were also excluded from the study due to concerns that their ongoing medical issues could potentially impact their mental state throughout the data collection process, and it would also generate biased results.

### 4.8 Data validation and quality control

PERI_DEP dataset has many attributes, such as age, gestational age, number of children, previous miscarriage, and many others. Data preprocessing becomes an essential step in data preparation for advanced analysis. In our study, data preprocessing includes data cleaning, normalization and solving class imbalance problem.

### 4.9 Data cleaning and normalization

Data cleaning and normalization are essential steps in the data preprocessing pipeline for several reasons. Firstly, data cleaning ensures the dataset is free from errors and inconsistencies, which could lead to inaccurate analysis and biased results. By identifying and rectifying these issues, data cleaning enhances the quality and reliability of the data, making it suitable for analysis and modeling. Normalizing the data ensures that each feature contributes equally to the analysis, leading to more robust and accurate models. Data cleaning and normalization play pivotal roles in ensuring data quality, enhancing analysis accuracy, and facilitating meaningful insights. The original PERI_DEP dataset contained 12 records with missing values. After removing these samples, there are 13,966 samples in the dataset. We have also normalized nominal features to numerical values using one-hot encoding. Table 4 shows statistical summary of original PERI_ DEP dataset.

**Table 4:** Statistical summary of Original PERI_DEP dataset.

	count	mean	std	min	25%	50%	75%	max
Age	14008	28.1	5.1	3	25	28	31	46
Gestational Age	14008	31.1	3.8	26	28	30	34	41
Number of sons	14008	1.1	1.1	0	0	1	2	6
Number of daughters	14008	1.1	1.2	0	0	1	2	6
Total Number of Children	14008	2.2	1.5	0	1	2	3	7
Little interest or pleasure in doing things	14008	1.4	1.1	0	0	1	2	3
Feeling down, depressed, or hopeless	14008	1.5	1.1	0	0	2	2	3
Trouble falling or staying asleep or sleeping too much	14008	1.3	1.1	0	0	1	2	3
Feeling tired or having little energy	14008	1.1	1.0	0	0	1	2	3
Poor appetite or overeating	14008	1.3	1.1	0	0	1	2	3
Feeling bad about yourself that you are a failure or have let yourself or your family down	14008	1.1	1.1	0	0	1	2	3
Trouble concentrating on things, such as reading the newspaper or watching television	14008	1.2	1.1	0	0	1	2	3
Moving or speaking so slowly that other people could have noticed	14008	1.2	1.1	0	0	1	2	3
Thoughts that you would be better off dead, or of hurting yourself	14008	1.1	1.1	0	0	1	2	3
EPDS-PHQ-Total-Score	14008	11.1	3.7	1	8	11	14	24

### 4.10 Dealing with class imbalance

There are a significantly higher number of instances in the original dataset labelled as depressed compared to those labelled as Non-depressed, thus creating a skewed data distribution. As the PERI_ DEP dataset is being used to develop a machine learning model for perinatal depression detection, the class imbalance problem can severely impact performance of the model by generating biased results. For solution of this problem and to avoid information loss, our preference is towards data augmentation through oversampling techniques. For this purpose, we have applied Generative Adversial Network (GAN) oversampling method. GAN generates synthetic samples derived from the original dataset [12]. The number of instances labelled as depressed was 7293 and those labelled as Non-depresses was 3903 in the dataset. We have applied GAN oversampling on 80% of the data samples by using CTGAN model in SVD python library. The size of the resulting dataset is 14586 with equal number of depressed and non-depressed records, thus creating a balanced PERI_ DEP dataset.

### 4.11 Re-use potential

The PERI_DEP dataset represents a significant advancement in the study of perinatal depression in Pakistan, addressing the critical issue of class imbalance in existing datasets. By employing a GAN oversampling technique, we have ensured a well-balanced dataset that enhances the accuracy and reliability of research outcomes. Our findings provide valuable insights into the socio-demographic differences between rural and urban women, highlighting the need for tailored interventions to address PND effectively.

The PERI_DEP dataset facilitates improved screening processes and customized interventions in hospital settings. It also supports researchers in identifying risk factors and developing targeted treatments. With its robust and representative sample, this comprehensive dataset has the potential to significantly impact public health strategies and elevate maternal mental health care standards in Pakistan.

## Limitations

A key limitation of our study is that our participants were recruited from a single geographical area in Pakistan, so our findings cannot be generalized to all women in the country during their perinatal period. However, our sample is likely to be reasonably representative of PND in the Punjab region of Pakistan. We aspire for the future to see an increased contribution of open datasets and meta-analysis to public health studies, enhancing scientific endeavours that can elevate humanity's living standards.

## Ethics Statement

The institutional review board at the Services Institute of Medical Sciences (Pakistan) approved this study with the reference IRB/2023/1155/SIMS. The primary method used for data collection in this study involved using standardized EPDS and PHQ-9 questionnaires covering socio-demographic and pregnancy-related information. For the PND study, the population consists of pregnant women and new mothers living in rural and urban areas of Pakistan who met the inclusion criteria. The study recruited participants who expressed interest and availability for participation.

## CRediT authorship contribution statement

**Amna Zafar:** Conceptualization, Data curation, Formal analysis, Investigation, Methodology, Writing – original draft, Writing – review & editing. **Beenish Ayesha Akram:** Conceptualization, Data curation, Formal analysis, Investigation, Methodology, Writing – original draft, Writing – review & editing. **Muhammad Wasim:** Conceptualization, Data curation, Formal analysis, Investigation, Methodology, Writing – original draft, Writing – review & editing. **Maham:** Data curation, Investigation, Methodology, Writing – original draft. **Ivan Miguel Pires:** Formal analysis, Funding acquisition, Investigation, Methodology, Project administration, Resources, Software, Writing – original draft, Writing – review & editing. **Paulo Jorge Coelho:** Formal analysis, Funding acquisition, Investigation, Methodology, Project administration, Resources, Software, Writing – original draft, Writing – review & editing.

## Declaration of Competing Interest

The authors declare that they have no known competing financial interests or personal relationships that could have appeared to influence the work reported in this paper.

## Data Availability

ZenodoPERI_DEP Dataset (Original data)

## Appendix Supplementary materials

Image, application 1
