# Supplementary material for: PERI_DEP: A dataset of mother's mental health in Pakistan
Source: Data Brief. 2025 May 7;60:111621. doi: 10.1016/j.dib.2025.111621 (PMC12151247; doi:10.1016/j.dib.2025.111621)
Supplement: Supplementary file 1 [file mmc1.zip › CodeBook.docx]

| **Socio-Demographic Variable** | **Meaning** | **Raw data file** | | **Augmented Data file** | | |
| --- | --- | --- | --- | --- | --- | --- |
|  |  | **Type** | **Unit** | **Feature Value** | **Encoding** | |
| Age | Age of a participating woman | Numeric | Years | Age | Years | |
| Gestational Age | Age of pregnancy from beginning of woman's last menstrual period (LMP) | Numeric | Weeks | Gestational Age | Weeks | |
| Number of Daughters | No. of daughters of a participant | Numeric | NA | Count | Numeric | |
| Total Number of Children | Total no. of children of participant | Numeric | NA | Count | Numeric | |
| Female Education | Educational level of a female participant | Ordinal | Uneducated | Uneducated | 0 | |
|  |  |  | Primary | Primary | 1 | |
|  |  |  | Middle | Middle | 2 | |
|  |  |  | Matric | Matric | 3 | |
|  |  |  | Intermediate | Intermediate | 4 | |
|  |  |  | Graduate | Graduate | 5 | |
| Husband Education | Educational level of husband of a female participant | Ordinal | Uneducated | Uneducated | | 0 |
|  |  |  | Primary | Primary | | 1 |
|  |  |  | Middle | Middle | | 2 |
|  |  |  | Matric | Matric | | 3 |
|  |  |  | Intermediate | Intermediate | | 4 |
|  |  |  | Graduation | Graduation | | 5 |
| Working Status | Housewife or Working lady | Nominal | House wife | House wife | | One-hot encoding |
|  |  |  | Working lady | Working lady | |  |
| Physical Health | Physical health of a participant | Nominal | Healthy | Healthy | | One-hot Encoding |
|  |  |  | Disabled | Disabled | |  |
| Sufficient money for basic needs | Financial status of a participant | Binary | Yes | Yes | | One-hot Encoding |
|  |  |  | No | No | |  |
| History of Miscarriage | Previous miscarriage of a participant | Binary | Yes | Yes | | One-hot Encoding |
|  |  |  | No | No | |  |
| Gravida  Primigravida  Multigravida | A pregnant Woman  A women pregnant for the first time  A woman pregnant more than once | Nominal | Primigravida | Primigravida | | One-hot Encoding |
|  |  |  | Multigravida | Multigravida | |  |
| Family System: Nuclear/Joint | Nuclear : Father, mother and children living separately in a house.  Joint : Grand Parents, married/unmarried siblings all living in one house | Nominal | Nuclear Family | Nominal | | One-hot Encoding |
|  |  |  | Joint Family |  |  |  |
| Male Gender Preference | Preference of a participant for male gender for child | Binary | Yes | Yes | | One-hot Encoding |
|  |  |  | No | No | |  |
| Relationship with In-laws | Level of relationship of a participant with in-laws | Nominal | Poor | Poor | | One hot Encoding |
|  |  |  | Moderate | Moderate | |  |
|  |  |  | Good | Good | |  |
| Current Appearance Acceptance | Acceptance of participant of her physical appearance | Binary | Yes | Yes | | One hot Encoding |
|  |  |  | No | No | |  |
