# Supplementary material for: PERI_DEP: A dataset of mother's mental health in Pakistan
Source: Data Brief. 2025 May 7;60:111621. doi: 10.1016/j.dib.2025.111621 (PMC12151247; doi:10.1016/j.dib.2025.111621)
Supplement: Supplementary file 1 [file mmc1.zip › CTGAN Data Generator.html]

CTGAN Data Generator


In [1]:

```
import pandas as pd
from sdv.single_table import CTGANSynthesizer
from sklearn.preprocessing import LabelEncoder
```

In [2]:

```
dataset = pd.read_csv('dataset.csv')
```

In [3]:

```
columns_to_strip = ['Female Education', 'Husband Education', 'Working Status', 
                    'Physical Health','Previous Miscarriage','Sufficient Money for Basic Needs', 
                    'Current Appereance Acceptance', 'Family System','Male Gender Preference', 'Relationship with Mother in-law']

# Strip whitespace from specific columns
dataset[columns_to_strip] = dataset[columns_to_strip].apply(lambda x: x.str.strip())
```

In [4]:

```
education_mapping = {'Uneducated': 0, 'Primary': 1, 'Middle': 2, 'Matric' : 3, 'Intermediate': 4, 'Graduation' : 5}
dataset['Female Education'] = dataset['Female Education'].map(education_mapping)
dataset['Husband Education'] = dataset['Husband Education'].map(education_mapping)
```

In [5]:

```
# Preprocess categorical variables
label_encoder = LabelEncoder()
for col in dataset.select_dtypes(include='object').columns:
    dataset[col] = label_encoder.fit_transform(dataset[col])
```

In [6]:

```
dataset = dataset.dropna(subset=["Age", "Thoughts that you would be better off dead, or of hurting yourself" , 
                                 "Moving or speaking so slowly that other people could have Noticed."])
```

In [7]:

```
X = dataset.iloc[:, :-1]
y = dataset.iloc[:, -1]
```

In [8]:

```
from sklearn.model_selection import train_test_split

X_train, X_test, y_train, y_test = train_test_split(X, y, test_size=0.2, random_state=42)
```

In [9]:

```
import numpy as np
X_y_test = np.hstack((X_test, y_test.values.reshape(-1,1)))
```

In [10]:

```
X_y_test.shape
```

Out[10]:

```
(2800, 26)
```

In [11]:

```
df_test_data = pd.DataFrame(X_y_test, columns=dataset.columns)
```

In [12]:

```
df_test_data.to_csv('test_data.csv', index=False)
```

In [13]:

```
y_train = y_train.values.reshape(-1, 1)
y_train.shape
```

Out[13]:

```
(11196, 1)
```

In [14]:

```
import numpy as np
X_y_train = np.hstack((X_train, y_train))
```

In [15]:

```
X_y_train
```

Out[15]:

```
array([[38., 31.,  1., ...,  2.,  0.,  0.],
       [26., 32.,  1., ...,  2.,  3.,  1.],
       [30., 29.,  0., ...,  0.,  1.,  0.],
       ...,
       [28., 29.,  3., ...,  2.,  2.,  1.],
       [36., 38.,  2., ...,  0.,  1.,  1.],
       [24., 28.,  1., ...,  2.,  0.,  1.]])
```

In [16]:

```
X_y_train.shape
```

Out[16]:

```
(11196, 26)
```

In [17]:

```
dataset.columns
```

Out[17]:

```
Index(['Age', 'Gestational Age', 'Number of sons', 'Number of daughters',
       'Total Number of Children', 'Gravida', 'Female Education',
       'Husband Education', 'Working Status', 'Physical Health',
       'Previous Miscarriage', 'Sufficient Money for Basic Needs',
       'Current Appereance Acceptance', 'Family System',
       'Male Gender Preference', 'Relationship with Mother in-law',
       'Little interest or pleasure in doing things',
       'Feeling down, depressed, or hopeless',
       'Trouble falling or staying sleep or sleeping too much',
       'Feeling tired or having little energy', 'Poor appetite or overeating',
       'Feeling badabout yourself that you are failure or have let yourself or your family down',
       'Trouble concentrating on things, such as reading the newspaper or watching television ',
       'Moving or speaking so slowly that other people could have Noticed.',
       'Thoughts that you would be better off dead, or of hurting yourself',
       'label'],
      dtype='object')
```

In [18]:

```
from sdv.metadata import SingleTableMetadata
metadata = SingleTableMetadata()

metadata.add_column('Age', sdtype='numerical')
metadata.add_column('Gestational Age', sdtype='numerical')
metadata.add_column('Number of sons', sdtype='numerical')
metadata.add_column('Number of daughters', sdtype='numerical')
metadata.add_column('Total Number of Children', sdtype='numerical')
metadata.add_column('Gravida', sdtype='numerical')
metadata.add_column('Female Education', sdtype='numerical')
metadata.add_column('Husband Education', sdtype='numerical')
metadata.add_column('Working Status', sdtype='numerical')
metadata.add_column('Physical Health', sdtype='numerical')
metadata.add_column('Previous Miscarriage', sdtype='numerical')
metadata.add_column('Sufficient Money for Basic Needs', sdtype='numerical')
metadata.add_column('Current Appereance Acceptance', sdtype='numerical')
metadata.add_column('Family System', sdtype='numerical')
metadata.add_column('Male Gender Preference', sdtype='numerical')
metadata.add_column('Relationship with Mother in-law', sdtype='numerical')
metadata.add_column('Little interest or pleasure in doing things', sdtype='numerical')
metadata.add_column('Feeling down, depressed, or hopeless', sdtype='numerical')
metadata.add_column('Trouble falling or staying sleep or sleeping too much', sdtype='numerical')
metadata.add_column('Feeling tired or having little energy', sdtype='numerical')
metadata.add_column('Poor appetite or overeating', sdtype='numerical')
metadata.add_column('Feeling badabout yourself that you are failure or have let yourself or your family down', sdtype='numerical')
metadata.add_column('Trouble concentrating on things, such as reading the newspaper or watching television ', sdtype='numerical')
metadata.add_column('Moving or speaking so slowly that other people could have Noticed.', sdtype='numerical')
metadata.add_column('Thoughts that you would be better off dead, or of hurting yourself', sdtype='numerical')

metadata.add_column('label', sdtype='categorical')
```

In [19]:

```
# Initialize the CTGAN model
ctgan = CTGANSynthesizer(metadata)
```

```
C:\Users\MuhammadWasim\AppData\Local\Programs\Python\Python312\Lib\site-packages\sdv\single_table\base.py:97: UserWarning: We strongly recommend saving the metadata using 'save_to_json' for replicability in future SDV versions.
  warnings.warn(
```

In [20]:

```
metadata
```

Out[20]:

```
{
    "METADATA_SPEC_VERSION": "SINGLE_TABLE_V1",
    "columns": {
        "Age": {
            "sdtype": "numerical"
        },
        "Gestational Age": {
            "sdtype": "numerical"
        },
        "Number of sons": {
            "sdtype": "numerical"
        },
        "Number of daughters": {
            "sdtype": "numerical"
        },
        "Total Number of Children": {
            "sdtype": "numerical"
        },
        "Gravida": {
            "sdtype": "numerical"
        },
        "Female Education": {
            "sdtype": "numerical"
        },
        "Husband Education": {
            "sdtype": "numerical"
        },
        "Working Status": {
            "sdtype": "numerical"
        },
        "Physical Health": {
            "sdtype": "numerical"
        },
        "Previous Miscarriage": {
            "sdtype": "numerical"
        },
        "Sufficient Money for Basic Needs": {
            "sdtype": "numerical"
        },
        "Current Appereance Acceptance": {
            "sdtype": "numerical"
        },
        "Family System": {
            "sdtype": "numerical"
        },
        "Male Gender Preference": {
            "sdtype": "numerical"
        },
        "Relationship with Mother in-law": {
            "sdtype": "numerical"
        },
        "Little interest or pleasure in doing things": {
            "sdtype": "numerical"
        },
        "Feeling down, depressed, or hopeless": {
            "sdtype": "numerical"
        },
        "Trouble falling or staying sleep or sleeping too much": {
            "sdtype": "numerical"
        },
        "Feeling tired or having little energy": {
            "sdtype": "numerical"
        },
        "Poor appetite or overeating": {
            "sdtype": "numerical"
        },
        "Feeling badabout yourself that you are failure or have let yourself or your family down": {
            "sdtype": "numerical"
        },
        "Trouble concentrating on things, such as reading the newspaper or watching television ": {
            "sdtype": "numerical"
        },
        "Moving or speaking so slowly that other people could have Noticed.": {
            "sdtype": "numerical"
        },
        "Thoughts that you would be better off dead, or of hurting yourself": {
            "sdtype": "numerical"
        },
        "label": {
            "sdtype": "categorical"
        }
    }
}
```

In [21]:

```
df = pd.DataFrame(X_y_train, columns=dataset.columns)
```

In [22]:

```
ctgan.fit(df)
```

In [23]:

```
df.label.value_counts()
```

Out[23]:

```
label
1.0    7293
0.0    3903
Name: count, dtype: int64
```

In [24]:

```
print(7293 - 3903) # samples to generate to balance the training dataset
```

```
3390
```

In [25]:

```
from sdv.sampling import Condition
```

In [26]:

```
conditions = Condition(num_rows=3390, column_values={'label': 0})
```

In [27]:

```
synthetic_data = ctgan.sample_from_conditions(
    conditions=[conditions]#,
    #output_file_path='synthetic_simulated_scenario.csv'
)
```

```
Sampling conditions: 100%|███████████████████████████████████████████████████████| 3390/3390 [00:00<00:00, 4198.33it/s]
```

In [28]:

```
synthetic_data
```

Out[28]:

|  | Age | Gestational Age | Number of sons | Number of daughters | Total Number of Children | Gravida | Female Education | Husband Education | Working Status | Physical Health | ... | Little interest or pleasure in doing things | Feeling down, depressed, or hopeless | Trouble falling or staying sleep or sleeping too much | Feeling tired or having little energy | Poor appetite or overeating | Feeling badabout yourself that you are failure or have let yourself or your family down | Trouble concentrating on things, such as reading the newspaper or watching television | Moving or speaking so slowly that other people could have Noticed. | Thoughts that you would be better off dead, or of hurting yourself | label |
| --- | --- | --- | --- | --- | --- | --- | --- | --- | --- | --- | --- | --- | --- | --- | --- | --- | --- | --- | --- | --- | --- |
| 0 | 23.0 | 34.0 | 0.0 | 1.0 | 1.0 | 0.0 | 2.0 | 4.0 | 1.0 | 1.0 | ... | 2.0 | 0.0 | 1.0 | 1.0 | 1.0 | 2.0 | 1.0 | 1.0 | 1.0 | 0.0 |
| 1 | 27.0 | 32.0 | 1.0 | 1.0 | 5.0 | 0.0 | 0.0 | 4.0 | 1.0 | 1.0 | ... | 0.0 | 0.0 | 1.0 | 0.0 | 2.0 | 0.0 | 1.0 | 1.0 | 0.0 | 0.0 |
| 2 | 28.0 | 37.0 | 1.0 | 1.0 | 2.0 | 0.0 | 4.0 | 3.0 | 0.0 | 1.0 | ... | 2.0 | 0.0 | 1.0 | 1.0 | 0.0 | 1.0 | 1.0 | 1.0 | 0.0 | 0.0 |
| 3 | 36.0 | 29.0 | 0.0 | 2.0 | 3.0 | 0.0 | 5.0 | 0.0 | 0.0 | 1.0 | ... | 0.0 | 0.0 | 1.0 | 1.0 | 3.0 | 0.0 | 0.0 | 1.0 | 0.0 | 0.0 |
| 4 | 36.0 | 26.0 | 1.0 | 1.0 | 3.0 | 0.0 | 1.0 | 4.0 | 1.0 | 1.0 | ... | 2.0 | 0.0 | 0.0 | 1.0 | 0.0 | 2.0 | 0.0 | 0.0 | 0.0 | 0.0 |
| ... | ... | ... | ... | ... | ... | ... | ... | ... | ... | ... | ... | ... | ... | ... | ... | ... | ... | ... | ... | ... | ... |
| 3385 | 22.0 | 29.0 | 2.0 | 1.0 | 4.0 | 0.0 | 4.0 | 1.0 | 0.0 | 1.0 | ... | 0.0 | 1.0 | 0.0 | 0.0 | 3.0 | 0.0 | 0.0 | 0.0 | 3.0 | 0.0 |
| 3386 | 22.0 | 29.0 | 0.0 | 1.0 | 1.0 | 2.0 | 1.0 | 3.0 | 1.0 | 1.0 | ... | 0.0 | 0.0 | 0.0 | 1.0 | 2.0 | 1.0 | 0.0 | 0.0 | 0.0 | 0.0 |
| 3387 | 20.0 | 38.0 | 0.0 | 1.0 | 0.0 | 2.0 | 4.0 | 0.0 | 1.0 | 1.0 | ... | 2.0 | 0.0 | 3.0 | 1.0 | 1.0 | 0.0 | 0.0 | 0.0 | 0.0 | 0.0 |
| 3388 | 35.0 | 38.0 | 0.0 | 2.0 | 4.0 | 0.0 | 1.0 | 3.0 | 1.0 | 1.0 | ... | 3.0 | 0.0 | 1.0 | 0.0 | 0.0 | 1.0 | 0.0 | 0.0 | 2.0 | 0.0 |
| 3389 | 28.0 | 29.0 | 0.0 | 2.0 | 2.0 | 0.0 | 4.0 | 3.0 | 0.0 | 1.0 | ... | 0.0 | 2.0 | 0.0 | 0.0 | 3.0 | 0.0 | 1.0 | 0.0 | 0.0 | 0.0 |

3390 rows × 26 columns

In [29]:

```
# only balance the training data from augmented samples
df.label.value_counts()
```

Out[29]:

```
label
1.0    7293
0.0    3903
Name: count, dtype: int64
```

In [30]:

```
augmented_data = pd.concat([df, synthetic_data], ignore_index=True)
```

In [31]:

```
augmented_data.shape
```

Out[31]:

```
(14586, 26)
```

In [32]:

```
augmented_data.label.value_counts()
```

Out[32]:

```
label
0.0    7293
1.0    7293
Name: count, dtype: int64
```

In [33]:

```
augmented_data.to_csv('augmented_data.csv', index=False)
```

In [ ]:

```

```
